# Impact of Long Working Hours and Shift Work on Perceived Unmet Dental Need: A Panel Study

**DOI:** 10.3390/ijerph18062939

**Published:** 2021-03-13

**Authors:** Hye-Eun Lee, Nam-Hee Kim, Tae-Won Jang, Ichiro Kawachi

**Affiliations:** 1 Korea Institute of Labor Safety and Health, Seoul 07023, Korea; heunn.lee@gmail.com; 2 Department of Social and Behavioral Sciences, Harvard T.H. Chan School of Public Health, Boston, MA 02115, USA; ikawachi@hsph.harvard.edu; 3 Department of Dental Hygiene, Wonju College of Medicine, Yonsei University, Wonju 26426, Korea; 4 Department of Occupational and Environmental Medicine, Hanyang University College of Medicine, Seoul 04763, Korea; om1024@hanmail.net

**Keywords:** work schedule tolerance, shift work schedule, dental health services

## Abstract

This study investigates whether workers with long working hours as well as shift workers perceive higher unmet dental care needs, and whether there is a gender difference in the associations. We used the Korea Health Panel (2009, 2011–2014) involving 20,451 person-wave observations from 5567 individuals. Perceived unmet dental care needs was defined when the participants reported that they perceived a need for dental treatment or check-up but had failed to receive dental care services during the past year. Fixed effects logit models were applied to examine how changes in weekly working hours or shift work status were linked to changes in perceived unmet dental needs within each individual. Among participants, 15.9–24.7% reported perceived unmet dental needs and the most common reason was time scarcity. We found that long working hours (>52 h/week) was significantly associated with perceived unmet dental needs due to time scarcity in both men (OR = 1.42, 95% CI 1.13–1.78) and women (OR = 1.35, 95% CI 1.03–1.79) compared workers working 40–52 h per week. Shift work was also a significant risk factor, but only in women (OR = 1.57, 95% CI 1.06–2.32). These findings provide evidence for labor policies to reduce working hours in order to improve access to dental care services.

## 1. Introduction

Unmet dental care needs occur when an individual fails to receive available and effective dental care that could have improved his/her oral health. Oral diseases that have not been treated appropriately can lead to deteriorated quality of life through not only physical problems but also social and psychological issues such as work function, appearance, and interpersonal relationships [1]. Perceived unmet dental care needs were much more frequently reported (26%) than unmet medical needs (8.8%) by Korean adults in the Korea National Health and Nutrition Examination Survey (KNHANES) in 2016 [2]. The prevalence of subjective unmet needs for dental examination and treatment in Korea is also much higher than the average of 28 European countries (4.0%) in 2019 [3]. Thus, identifying and intervening on risk factors for perceived unmet dental care needs is a priority in Korea.

According to a previous Korean study, 9.4% of workers per year experienced sickness absence (including early retirement from work), and almost half of those surveyed reported that dental disease negatively affected their work performance [4]. Failure of access to timely dental care could lead not only to poor oral health but also to productivity losses.

Andersen’s behavioral model, which has been widely used in studies of health services utilization, focuses on predisposing, enabling, and need variables in terms of predicting the use of health services [5]. Variables in the predisposing domain include demographic characteristics, health beliefs, and social structures. The enabling domain includes personal/family resources, while the need domain includes perceived and evaluated need [6]. Previous Korean studies have found a correlation between perceived unmet dental care needs and (1) predisposing variables such as younger age, female gender, marital status, and low education level [2,7]; (2) enabling variables such as income, engagement in precarious work, unemployment, medical aid as a type of insurance, and long working hours [2,8,9,10,11], and (3) need factors such as poor oral health status [12].

As different policy approaches are needed to reduce unmet health care needs for different reasons, it is necessary to understand the contributions of specific enabling factors. South Korea is notorious for long working hours. In 2019, the annual average working hours in Korea was 1967 h, which was 241 h longer than the average annual working hours reported for the Organization for Economic Cooperation and Development (OECD) countries as a whole [13]. Non-standard working hours involving work hours outside typical or standard working hours has become very common worldwide [14]. According to a survey by the Korean government, around 15% of all employed workers were engaged in nonstandard work schedules in 2011, which was similar to other Western countries [15]. Long working hours can be an important cause of time scarcity or “time poverty” [16,17]. Non-standard working hours (such as shift work) can also directly intrude on socially valuable time [14]. Thus time is an important resource like income for people to stay healthy, and to maintain satisfactory social and family life [16]. Time pressure related to work (20.9%) was the second most common reason given for perceived unmet dental needs in Korea, after financial reasons (41.4%) [10].

The majority of previous Korean studies on unmet dental needs have focused on socioeconomic inequalities rather than time-related factors [2,8,9,10]. However, socially deprived people and time poor do not always coincide. For example, long working hours were more prevalent among male permanent workers and high school educated workers in Korea [18]. Although a previous study investigated the association between long working hours and perceived unmet dental needs, the study design was cross-sectional study and the reason for perceived unmet dental needs was not considered in the outcome measure [11]. In addition, little attention has been paid to working hour arrangements including shiftwork. We hypothesized that workers with long working hours or workers engaged in shift work experience more difficulties in utilizing dental care due to time scarcity. In addition, we hypothesized that the impact of working hours on unmet dental care will vary according to gender. Women are more likely to suffer from time poverty because they spend more time on unpaid domestic labor than men [17]. Therefore, this study sought to investigate the impact of long working hours and shift work status on perceived unmet dental needs by gender, using representative panel data in Korea.

## 2. Materials and Methods

### 2.1. Study Population

Data used in this study were derived from the Korean Health Panel Study (KHPS) conducted in 2009 and 2011–2014 by the Korean Institute for Health and Social Affairs in conjunction with the National Health Insurance Service. The KHPS is a national panel survey on a representative sample of South Korean households. The survey employed a two-stage random sampling design based on the Population and Housing Census. Trained medical staff interviewed participants using a computer-assisted personal interviewing (CAPI) technique. 

**Figure 1:**
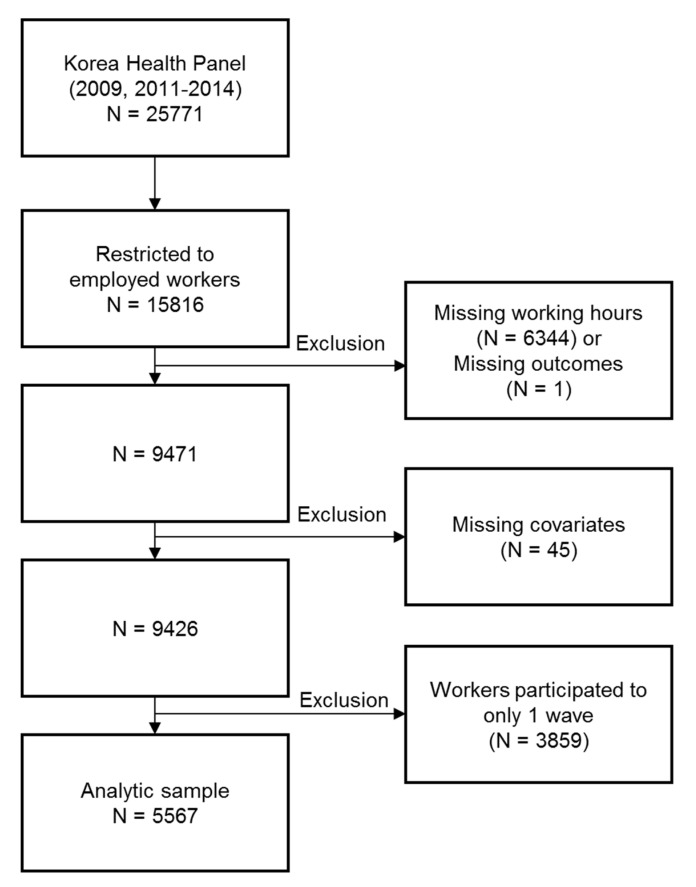
Flow chart of the participants selection.

We used data of 5 waves where working hours and shift work status were measured. We restricted the subjects to employed workers, and individuals with missing values were excluded. To leverage the panel data, we excluded individuals who participated in only one wave. As a result, a total of 20,451 person-wave observations from 5567 workers remained in the analysis. The analytic sample selection process is presented in Figure 1.

### 2.2. Measurement

#### 2.2.1. Perceived Unmet Dental Need

Perceived unmet dental care needs was defined if the participants responded “yes” to the question “Did you ever fail to receive dental care services over the past year, even when there was a need for treatment or check-up?”. The reasons were also surveyed so we specified perceived unmet dental needs due to time scarcity and due to financial issues. 

#### 2.2.2. Working Hours

The number of hours worked per week and shift work was surveyed as of 31st of December last year in each wave of survey. We classified the weekly working hours into three categories (1) <40 h/week, (2) 40–52 h/week, and (3) >52 h/week based on the fact that Labor Standards Act in Korea defines 40 h per week as standard working hours and 52 h per week as the maximum permissible working hours. 

Shift work was defined if the individuals responded “no” to the question “Do you work usually in the daytime (06:00–18:00)?”. Shift workers were further classified into: (1) evening work, (2) night work, (3) day and night regular shift work, (4) 24-h shift work, (5) split shift, and (6) irregular shift work. 

#### 2.2.3. Covariates

Potential confounding factors included age, sex, education, marital status, household income, occupation, and employment status. We used household income quintile categories provided in the data, which was calculated using square root scale. The occupation was classified into 9 categories according to the Korean standard classification of occupation. Employment status was categorized into 2 groups, permanent or regular workers vs. temporary or daily workers. 

### 2.3. Statistical Analysis

We used fixed effects (FE) logit models to examine the hypotheses that working hours affect perceived unmet dental needs. Because the association between working hours and unmet dental needs is likely confounded by unmeasured third variables which cannot be easily controlled for, fixed effects models were considered as the most appropriate model choice [19]. In this model, unobserved confounding effects from time-invariant variables can be eliminated because the analysis examines how changes in working hours or shift work were associated with changes in perceived unmet dental needs within each individual over time. The associations of predictors with perceived unmet dental needs in the present study were defined to occur during the same period (no time lag), since working hours or shiftwork are likely to have an immediate impact on unmet dental needs, if there is an association. We controlled for several time-varying factors by including relevant covariates such as age, marital status, education, household income, employment status, and occupation into the fixed effects models. All FE models were stratified by gender. The significance level for statistical analyses was *p* < 0.05 using a two-tailed test. SAS version 9.4 (SAS Institute, Cary, NC, USA) was used for statistical analysis.

## 3. Results

The unbalanced panel data for analysis included 20,451 person-wave observations from 5567 participants (Table 1). The prevalence of participants who reported perceived unmet dental needs were 24.7% in 2009, 16.7% in 2011, 16.7% in 2012, 18.1% in 2013, and 15.9% in 2014. The proportion of workers who responded that time scarcity was the reason for perceived unmet dental needs were 10.7% in 2009, 6.4% in 2011, 6.8% in 2012, 7.7% in 2013, and 6.9% in 2014. The proportion of workers working more than 52 h per week gradually decreased from 30.7% in 2009 to 21.4% in 2014. Shift work also declined from 15.0% in 2009 to 11.5% in 2011 and then remained stable thereafter.

Average weekly working hours and prevalence of shift work by characteristics of subjects are presented in Supplementary Table S1. Working hours were longer among men (50.1 ± 14.1 h/week) than women (42.5 ± 15.7 h/week) and shift work was more prevalent in men (17.5%) than women (11.8%) as well. Agricultural, forestry, and fishery workers (53.3 ± 17.2 h/week) and plant, machine operators, and assemblers (52.2 ± 13.4 h/week) worked the longest hours. Shift work was most common among plant, machine operators, and assemblers (33.6%) and clerks (31.7%). 

The results of FE models for the association between working hours and total perceived unmet dental needs (regardless of the reasons) are shown in Table 2. In men, shift work was associated with perceived unmet dental needs (odds ratio (OR) = 1.48, 95% confidence interval (CI) 1.14–1.91). In addition, part-time or day workers showed a higher risk (OR = 1.25, 95% CI 1.01–1.55) for perceived unmet dental needs compared to full-time or regular workers. In women, the lowest income group was associated with perceived unmet dental needs (OR = 1.60, 95% CI 1.00–2.56).

Table 3 shows the results of FE models for the association between working hours and perceived unmet dental needs due to time scarcity. Long working hours (>52 h/week) showed a significant association with perceived unmet dental needs stemming from time scarcity among both men (OR = 1.42, 95% CI 1.13–1.78) and women (OR = 1.35, 95% CI 1.03–1.79). Shift work was identified as a significant risk factor only in women (OR = 1.57, 95% CI 1.06–2.32). In women, the lowest income group showed a significantly lower risk of perceived unmet dental needs due to time scarcity (OR = 0.45, 95% CI 0.21–0.93). The results of FE models for perceived unmet dental needs stemming from financial issues are shown in Supplementary Table S2.

## 4. Discussion

The results of this study support an association between long working hours (>52 h/week) and perceived unmet dental needs due to time scarcity. Results also suggest that the effects of shift work on perceived unmet dental needs differed for men and women; a significant association was found only in women.

The impact of long working hours on perceived unmet dental needs due to time scarcity is in line with previous Korean studies. A study using data of the Korea National Health and Nutrition Examination Survey (KNHANES) reported that participants working long hours (>60 h/week) experienced more perceived unmet dental needs (OR = 1.54, 95% CI 1.17–2.02) compared to participants who do not work (who reported “zero” working hours) among Korean men [11]. Another cross-sectional study using the KNHANES found a significant association between long working hours (50–59 and ≥60 vs. 30–39 h/week) and perceived unmet healthcare needs with a dose–response relationship in both men and women [20]. However, previous studies of the association between working hours and unmet needs may be biased by unobserved confounding factors that influence both working hours and dental care utilization. Our fixed-effects analysis provides a more secure basis for causal inference, given that the approach differences out all time-invariant observed and unobserved confounding factors. 

The finding of an association between long working hours and perceived unmet dental needs due to time scarcity is not surprising, given that seeing the dentist requires time off work. The Labor Standards Act in Korea includes no regulation about paid sick leave, so it is not easy for workers to take a day (or half-day) off for getting dental care. Receiving preventive care would be even more neglected since it is not urgent. 

A few studies have investigated the impact of shift work on unmet needs. A previous Korean study found no association between shift-work and unmet healthcare needs in either men or women [21]. However, the outcome used in the previous study was total unmet needs (without considering the reasons for unmet needs). Interestingly, results in the present study showed the impact of shift work on perceived unmet dental needs due to time scarcity, but only in women. Although the evidence is somewhat inconsistent, many researchers have claimed that shiftwork has a worse impact on women than on men in terms of health, sleep, and fatigue, [22]. A plausible reason for this gender disparity is that women are more likely to have household and family responsibilities compared to men so that women workers are under a double burden from work and family roles [17,18]. Shift work itself can be related to low work-time control. A recent Finnish study showed that low control over daily hours was reported by 54% of shift workers while 26% of non-shift workers reported low control [23]. In addition, the Time use survey in Korea revealed that in dual-income families, men spent an average of 37 min, whereas women spent an average of 3 h and 20 min per day on household works and caring for families in 2009 [24]. Furthermore, the support systems for family care have remained insufficient for shift workers in Korea. For example, 20.5% of hospitals had childcare facilities for nurses, but only 4.8% of the facilities in hospitals operate 24 h a day for shift workers [25]. Considering that dental care often needs frequent visits over a long time, workers who have low control over their daily work schedules might easily give up on starting treatment. Even though shift-workers’ working schedules may not overlap with dentist service hours, women shift-workers may be too busy performing family responsibilities to seek dental care outside their work hours. In order to solve the problem of time scarcity among women engaged in shift work, a support system for family care is also needed along with improving the working conditions. 

In South Korea, although dental service coverage is limited, basic dental care such as treatment for dental caries and periodontal disease are covered by National health insurance with 30–50% out-of-pocket expenses. However, household income level also showed a significant association with total perceived unmet dental needs (including financial reasons and time scarcity)—but only in women. Income is a well-established predisposing factor for unmet dental needs according to previous Korean studies [2,9,10] and many studies reported that women reported more perceived unmet dental needs than men [7,9,10]. However, the evidence on gender differences in the association between income and unmet dental care has been inconsistent. A previous study using KNHANES reported a worse impact of low income on perceived unmet dental needs in women than in men [26], whereas other studies found no significant gender differences [11,27]. Our results suggest that women lacking financial resources might be more unlikely to utilize dental care than men. 

In the present study, the prevalence of perceived unmet dental needs was around 16–25%. The most common reason was time scarcity, which was reported by about 40%, while financial barriers were reported by about 20%. Our findings are in contrast to a previous study using data from KNHANES (2007–2009), in which 43.9% of Korean adults reported perceived unmet dental needs, and financial difficulties was the most common reason [10]. This discrepancy may result from the fact that our study sample was made up of employed workers and not the general population. This also suggests the necessity to pay attention to the different reasons for unmet dental needs in different populations to plan effective policies.

The present study has some limitations. First, our outcome was based on self-report. Therefore, perceived unmet dental needs may not correlate with clinically assessed needs. However, self-reported unmet needs may better reflect the individuals’ subjective assessments of their oral health status [28]. According to a previous report comparing self-report and clinically diagnosed unmet dental needs, the specificity of self-reported unmet dental needs was highly specific (>90%), i.e., few false-positives, although it was not very sensitive—i.e., more false negatives [29]. Therefore, there is a possibility of underestimation of unmet dental needs in the study sample, but as the associations between working hours and perceived unmet dental needs were analyzed by a within-individual design, we can eliminate between-individual differences in health perception as a source of bias. Second, because the time frame of measurement of working hours and perceived unmet dental needs were simultaneous, reverse causation cannot be ruled out. However, it is unlikely that workers with unmet dental needs (due to time scarcity) increased their working hours or chose shift work. Therefore, it seems reasonable to interpret that working hours affect unmet dental needs rather than the reverse. Third, we could not control for individuals’ dental health status due to lack of information. Oral health status probably affects participants’ access to dental care and might influence their selection of working schedules. For example, individuals with severe dental disease may experience unmet dental needs more easily and may avoid demanding work schedules such as long working hours or night shift work.

Despite these limitations, the present study is the first study to investigate the associations between working hours and shift work status and perceived unmet dental care needs using longitudinal panel data. This within-individual analysis approach allowed us to control for unmeasured time-invariant confounders. In addition, we found gender differences in the impact of shift work on perceived unmet dental needs. Our findings are useful in suggesting the direction of policies to improve the access to dental care when the target population is workers. 

## 5. Conclusions

We found that the most important reason for perceived unmet dental needs among Korean workers was time scarcity. We also found that shift work had a worse impact on women’s perceived unmet dental needs. The findings suggest that reducing working hours can be one of the policies to improve access to health care service for Korean workers and that addressing time poverty of women engaged in shift work should be a policy priority. 

## Figures and Tables

**Table 1:** General characteristics of the subjects.

	2009	2011	2012	2013	2014
	N = 3398	N = 4553	N = 4467	N = 4212	N = 3785
Perceived unmet dental need	838 (24.7)	759 (16.7)	746 (16.7)	763 (18.1)	603 (15.9)
due to financial issue	137 (4.0)	144 (3.2)	177 (4.0)	161 (3.8)	79 (2.1)
due to time scarcity	365 (10.7)	290 (6.4)	303 (6.8)	326 (7.7)	262 (6.9)
Sex (male)	1943 (57.2)	2631 (57.8)	2552 (57.1)	2399 (57.0)	2168 (57.3)
Age (year), mean ± SD	43.1 ± 11.6	44.2 ± 12.1	44.7 ± 12.2	45.2 ± 12.4	46.2 ± 12.4
Marital status					
Single or living alone	899 (26.5)	1281 (28.1)	1269 (28.4)	1229 (29.2)	1086 (28.7)
Married or living with partner	2499 (73.5)	3272 (71.9)	3198 (71.6)	2983 (70.8)	2699 (71.3)
Education					
Lower than high school diploma	694 (20.4)	863 (19.0)	815 (18.2)	743 (17.6)	664 (17.5)
High school diploma	1750 (51.5)	2392 (52.5)	2347 (52.5)	2227 (52.9)	1997 (52.8)
Undergraduate and higher	954 (28.1)	1298 (28.5)	1305 (29.2)	1242 (29.5)	1124 (29.7)
Household income					
Q1 (lowest)	158 (4.7)	215 (4.7)	192 (4.3)	193 (4.6)	170 (4.5)
Q2	508 (15.0)	677 (14.9)	718 (16.1)	678 (16.1)	588 (15.5)
Q3	751 (22.1)	1122 (24.6)	1074 (24.0)	992 (23.6)	913 (24.1)
Q4	970 (28.6)	1230 (27.0)	1189 (26.6)	1160 (27.5)	1032 (27.3)
Q5 (highest)	1011 (29.8)	1309 (28.8)	1294 (29.0)	1189 (28.2)	1082 (28.6)
Employment status					
Full-time, regular	1950 (57.4)	2478 (54.4)	2341 (52.4)	2221 (52.7)	2010 (53.1)
Part-time, day worker, etc.	1448 (42.6)	2075 (45.6)	2126 (47.6)	1991 (47.3)	1775 (46.9)
Occupation					
Legislators, senior officials, and managers	159 (4.7)	251 (5.5)	242 (5.4)	242 (5.8)	194 (5.1)
Professionals	708 (20.8)	906 (19.9)	892 (20.0)	834 (19.8)	766 (20.2)
Technicians and associate professionals	505 (14.9)	647 (14.2)	621 (13.9)	608 (14.4)	546 (14.4)
Clerks	284 (8.4)	405 (8.9)	407 (9.1)	363 (8.6)	340 (9.0)
Service and sale workers	268 (7.9)	346 (7.6)	348 (7.8)	332 (7.9)	301 (8.0)
Agricultural, forestry, and fishery workers	16 (0.5)	26 (0.6)	33 (0.7)	21 (0.5)	18 (0.5)
Craft and related trades workers	433 (12.7)	576 (12.7)	587 (13.1)	554 (13.2)	497 (13.1)
Plant, machine operators, and assemblers	342 (10.1)	422 (9.3)	411 (9.2)	384 (9.1)	333 (8.8)
Elementary occupations	683 (20.1)	974 (21.4)	926 (20.7)	874 (20.8)	790 (20.9)
Weekly working hour					
<40	549 (16.2)	647 (14.2)	650 (14.6)	597 (14.2)	545 (14.4)
40–52	1807 (53.2)	2671 (58.7)	2709 (60.6)	2677 (63.6)	2431 (64.2)
>52	1042 (30.7)	1235 (27.1)	1108 (24.8)	938 (22.3)	809 (21.4)
Shift work (yes)	511 (15.0)	523 (11.5)	512 (11.5)	482 (11.4)	428 (11.3)

Values are presented as number (%). SD, standard deviation.

**Table 2:** Results from fixed effect logit models for the association between working hours and perceived unmet dental needs.

	Men			Women		
	OR	95% CI	*p*	OR	95% CI	*p*
Weekly working hour						
<40	1.28	0.97–1.70	0.08	1.16	0.91–1.46	0.23
40–52	1.00			1.00		
>52	1.06	0.90–1.25	0.48	0.83	0.66–1.06	0.13
Shift work						
No	1.00					
Yes	1.48	1.14–1.91	0.003	1.24	0.92–1.68	0.16
Age	0.91	0.88–0.95	<0.0001	0.87	0.83–0.90	<0.0001
Marital status						
Single or living alone	1.00			1.00		
Married or living with partner	0.90	0.51–1.58	0.71	0.69	0.40–1.21	0.20
Education						
Lower than high school diploma	-		0.97	-		0.97
High school diploma	1.02	0.26–4.01	0.98	1.05	0.34–3.27	0.93
Undergraduate and higher	1.00			1.00		
Household income						
Q1 (lowest)	1.02	0.63–1.63	0.94	1.60	1.00–2.56	0.05
Q2	0.97	0.70–1.36	0.87	1.21	0.83–1.74	0.32
Q3	0.95	0.71–1.26	0.70	0.94	0.68–1.29	0.70
Q4	0.99	0.78–1.25	0.90	1.16	0.89–1.52	0.28
Q5 (highest)	1.00			1.00		
Employment status						
Full-time, regular	1.00			1.00		
Part-time, day worker, etc.	1.25	1.01–1.55	0.04	1.25	0.99–1.57	0.06
Occupation						
Legislators, senior officials, and managers	1.00			1.00		
Professionals	1.12	0.66–1.90	0.66	1.42	0.48–4.20	0.53
Technicians and associate professionals	1.33	0.80–2.21	0.26	1.46	0.49–4.30	0.49
Clerks	1.69	0.79–3.58	0.17	0.97	0.33–2.91	0.96
Service and sale workers	0.84	0.45–1.56	0.58	1.22	0.42–3.53	0.72
Agricultural, forestry, and fishery workers	0.99	0.27–3.62	0.99	0.46	0.06–3.83	0.47
Craft and related trades workers	1.12	0.69–1.82	0.66	1.29	0.37–4.46	0.69
Plant, machine operators, and assemblers	0.83	0.48–1.42	0.49	0.83	0.22–3.08	0.78
Elementary occupations	1.02	0.59–1.76	0.94	1.18	0.40–3.53	0.76

**Table 3:** Results from fixed effect logit models for the association between working hours and perceived unmet dental need due to time scarcity.

	Men			Women		
	OR	95% CI	*p*	OR	95% CI	*p*
Weekly working hour						
<40	0.79	0.46–1.35	0.39	0.57	0.40–0.81	0.002
40–52	1.00			1.00		
>52	1.42	1.13–1.78	0.003	1.35	1.03–1.79	0.03
Shift work						
No	1.00					
Yes	1.01	0.67–1.55	0.95	1.57	1.06–2.32	0.02
Age	0.91	0.87–0.96	0.0007	0.91	0.86–0.96	0.0008
Marital status						
Single or living alone	1.00			1.00		
Married or living with partner	0.58	0.24–1.37	0.21	1.19	0.57–2.50	0.65
Education						
Lower than high school diploma	-		0.98	0.22	0.02–3.17	0.26
High school diploma	0.09	0.01–0.84	0.03	0.31	0.06–1.65	0.17
Undergraduate and higher	1.00			1.00		
Household income						
Q1	1.13	0.49–2.58	0.77	0.45	0.21–0.93	0.03
Q2	0.93	0.56–1.55	0.78	0.66	0.41–1.06	0.08
Q3	1.10	0.72–1.66	0.67	0.86	0.58–1.29	0.47
Q4	1.07	0.77–1.48	0.69	1.07	0.78–1.47	0.67
Q5	1.00			1.00		
Employment status						
Full-time, regular	1.00			1.00		
Other	1.14	0.83–1.56	0.43	0.70	0.53–0.92	0.01
Occupation						
Legislators, senior officials, and managers	1.00			1.00		
Professionals	0.95	0.50–1.82	0.88	0.87	0.19–3.96	0.85
Technicians and associate professionals	1.25	0.64–2.43	0.52	0.85	0.19–3.82	0.83
Clerks	2.54	0.84–7.69	0.10	1.33	0.29–6.02	0.71
Service and sale workers	0.87	0.33–2.29	0.77	1.12	0.25–5.02	0.89
Agricultural, forestry, and fishery workers	1.36	0.23–8.01	0.74	–		0.98
Craft and related trades workers	1.49	0.74–3.01	0.27	2.43	0.45–13.12	0.30
Plant, machine operators, and assemblers	1.75	0.83–3.68	0.14	0.87	0.16–4.78	0.87
Elementary occupations	1.21	0.54–2.71	0.65	1.32	0.29–6.12	0.72

## Data Availability

The data that support the findings of this study are available on request from the Korea Health Panel Study (https://www.khp.re.kr) (accessed on 11 January 2021).

## Supplementary Materials

The following are available online at https://www.mdpi.com/1660-4601/18/6/2939/s1, Table S1: Average weekly working hours and prevalence of shift work by population characteristics in 2009 (N = 3398), Table S2: Results from fixed effect logit models for the association between working hours and unmet dental need due to financial issue.
