# Supplementary material for: Impact of Long Working Hours and Shift Work on Perceived Unmet Dental Need: A Panel Study
Source: Int J Environ Res Public Health. 2021 Mar 13;18(6):2939. doi: 10.3390/ijerph18062939 (PMC8001453; doi:10.3390/ijerph18062939)
Supplement: Supplementary file 1 [file ijerph-18-02939-s001.pdf]

Table S1. Average weekly working hours and prevalence of shift work by population characteristics in 2009 (N=3398)

	Weekly working hours (mean±SD)	Shift work (%)
Perceived unmet dental need		
No	47.1±14.9	15.2
Yes	46.0±16.1	14.7
Unmet dental need due to financial issue		
No	47.0±15.1	15.0
Yes	43.0±18.8	15.3
Unmet dental need due to time scarcity		
No	46.5±15.4	15.6
Yes	49.4±13.7	10.7
Sex		
Men	50.1±14.1	17.5
Women	42.5±15.7	11.8
Age (y)		
<30	46.9±14.8	19.0
30-39	47.6±14.2	13.1
40-49	46.9±14.5	12.9
50-59	46.0±15.0	14.3
≥60	46.1±20.7	23.6
Marital status		
Single or living alone	45.9±15.3	17.7
Married or living with partner	47.2±15.2	14.1
Education		
Lower than high school diploma	46.0±18.0	15.9
High school diploma	47.5±14.9	17.7
Undergraduate and higher	46.2±13.5	9.6
Household income		
Q1 (Lowest)	38.8±20.0	9.5
Q2	46.2±17.4	17.3
Q3	47.6±16.3	14.1
Q4	48.4±14.4	17.7
Q5 (Highest)	46.3±12.5	12.9
Employment status		
Full-time, regular	49.5±11.0	14.8
Part-time, day worker, etc.	43.3±19.0	15.4
Occupation		
Legislators, senior officials, and managers	47.0±9.8	6.9
Professionals	45.0±13.6	10.0
Technicians and associate professionals	46.0±9.5	5.2
Clerks	50.2±20.9	31.7
Service and sale workers	44.9±18.2	13.1
Agricultural, forestry, and fishery workers	53.3±17.2	18.8
Craft and related trades workers	49.1±13.0	10.4
Plant, machine operators, and assemblers	52.2±13.4	33.6
Elementary occupations	44.3±18.1	16.8

Weekly working hour		
<40		9.3
40-52		11.5
>52		24.3
Shift work		
No	45.5±13.9	
Yes	54.2±19.8	

Table S2. Results from Fixed effect logit models for the association between working hours and perceived unmet dental need due to financial issue

	Men		Women	
	OR	95% CI	OR	95% CI
Weekly working hour				
<40	0.92	0.55 - 1.55	1.69	1.13 - 2.53
40-52	1.00		1.00	
>52	0.90	0.61 - 1.34	1.06	0.66 - 1.69
Shift work				
No	1.00			
Yes	1.39	0.80 - 2.39	0.92	0.51 - 1.64
Age	0.94	0.86 - 1.02	0.92	0.85 - 1.00
Marital status				
Single or living alone	1.00		1.00	
Married or living with partner	0.36	0.06 - 2.00	0.87	0.30 - 2.54
Education				
Lower than high school diploma	-		-	
High school diploma	-		1.63	0.11 - 23.67
Undergraduate and higher	1.00		1.00	
Household income				
Q1 (lowest)	2.79	0.98 - 7.96	3.34	1.29 - 8.65
Q2	2.33	0.94 - 5.77	2.07	0.88 - 4.89
Q3	1.94	0.84 - 4.46	1.71	0.76 - 3.85
Q4	1.61	0.73 - 3.56	1.55	0.75 - 3.20
Q5 (highest)	1.00		1.00	
Employment status				
Full-time, regular	1.00		1.00	
Other	1.35	0.83 - 2.22	1.31	0.80 - 2.14
Occupation				
Legislators, senior officials, and managers	1.00		1.00	
Professionals	0.85	0.12 - 6.07	0.17	0.02 - 1.58
Technicians and associate professionals	3.54	0.84 - 14.97	0.46	0.06 - 3.63
Clerks	4.16	0.67 - 25.95	0.22	0.03 - 1.71

Service and sale workers	1.16	0.24 - 5.65	0.50	0.06 - 3.87
Agricultural, forestry, and fishery workers	-		0.26	0.01 - 8.67
Craft and related trades workers	1.57	0.46 - 5.37	0.14	0.01 - 1.61
Plant, machine operators, and assemblers	2.20	0.55 - 8.77	0.15	0.01 - 2.19
Elementary occupations	1.71	0.47 - 6.25	0.21	0.03 - 1.66
